# Muramyl Dipeptide Induces NOD2-Dependent Ly6C^high^ Monocyte Recruitment to the Lungs and Protects Against Influenza Virus Infection

**DOI:** 10.1371/journal.pone.0036734

**Published:** 2012-05-09

**Authors:** François Coulombe, Stéphanie Fiola, Shizuo Akira, Yvon Cormier, Jean Gosselin

**Affiliations:** 1 Centre Hospitalier Universitaire de Québec Research Center and Department of Molecular Medicine, Faculty of Medicine, Laval University, Quebec, Canada; 2 Laboratory of Host Defense, World Premier International Immunology Frontier Research Center and Department of Host Defense, Research Institute for Microbial Diseases, Osaka University, Osaka, Japan; 3 Institut Universitaire de Cardiologie et de Pneumologie du Québec and Department of Medicine, Faculty of Medicine, Laval University, Quebec, Canada; University of São Paulo, Brazil

## Abstract

Bacterial peptidoglycan-derived muramyl dipeptide (MDP) and derivatives have long-recognized antiviral properties but their mechanism of action remains unclear. In recent years, the pattern-recognition receptor NOD2 has been shown to mediate innate responses to MDP. Here, we show that MDP treatment of mice infected with Influenza A virus (IAV) significantly reduces mortality, viral load and pulmonary inflammation in a NOD2-dependent manner. Importantly, the induction of type I interferon (IFN) and CCL2 chemokine was markedly increased in the lungs following MDP treatment and correlated with a NOD2-dependent enhancement in circulating monocytes. Mechanistically, the protective effect of MDP could be explained by the NOD2-dependent transient increase in recruitment of Ly6C^high^ “inflammatory” monocytes and, to a lesser extent, neutrophils to the lungs. Indeed, impairment in both Ly6C^high^ monocyte recruitment and survival observed in infected *Nod2*-/- mice treated with MDP was recapitulated in mice deficient for the chemokine receptor CCR2 required for CCL2-mediated Ly6C^high^ monocyte migration from the bone marrow into the lungs. MDP-induced pulmonary monocyte recruitment occurred normally in IAV-infected and MDP-treated *Ips-1*-/- mice. However, IPS-1 was required for improved survival upon MDP treatment. Finally, mycobacterial *N*-glycolyl MDP was more potent than *N*-acetyl MDP expressed by most bacteria at reducing viral burden while both forms of MDP restored pulmonary function following IAV challenge. Overall, our work sheds light on the antiviral mechanism of a clinically relevant bacterial-derived compound and identifies the NOD2 pathway as a potential therapeutic target against IAV.

## Introduction

Extending from the concept of vaccination employed by Edward Jenner more than two centuries ago, early studies performed with Freund's complete adjuvant have contributed to establish the notion that microbial organisms could be decomposed and still retain immunostimulatory properties. Freund's original preparation consisting of heat-killed mycobacterial cells suspended in mineral oil, could indeed be recapitulated by the cell wall fraction of mycobacteria and related organisms [1]. The smallest bacterial cell wall constituent that retained immunogenic properties was identified as the peptidogylcan *N*-acetylmuramyl-L-alanyl-D-isoglutamine substructure, commonly referred to as MDP [2]. This molecule was synthesized and found to have antibacterial, antiviral and tumoricidal activity [3]–[5]. Since then, an impressive number of MDP derivatives were generated, some of which have reached clinical trials for the treatment of infectious diseases and cancer or as vaccine adjuvant [6]–[8]. However, the mechanism of action of MDP still remains unknown.

In 2003, the NOD-like receptor NOD2 was identified as the host PRR responsible for MDP recognition [9]. Upon MDP sensing, NOD2 activates mitogen-activated protein (MAP) kinases as well as the transcription factors NF-κB and IRF5 via the RIP2 adaptor molecule leading to the secretion of inflammatory mediators, chemokines and type I IFNs [10]–[12]. Since the discovery of NOD2, it has been assumed, but never formerly demonstrated, that the antiviral activity of MDP requires this receptor. However, a growing body of evidence suggests that NOD2 triggering could effectively be beneficial against several viral infections including IAV. First, stimulation of macrophages with MDP is known to induce the production of type I IFNs [12], which are required to control IAV infection [13], [14]. In addition, NOD2 has been shown to play a role in the recognition of viral single-stranded RNA (ssRNA) and to activate antiviral innate immunity via the IPS-1 adaptor molecule [15]. A recent study also shows that NOD2 promotes the CCL2-CCR2-dependent recruitment of Ly6C^high^ monocytes in the intestine of *Citrobacter rodentium*-infected mice [16]. In parallel, Seo and colleagues have demonstrated an indispensable and protective role for type I IFN signaling in the differentiation and recruitment of Ly6C^high^ monocytes during IAV infection [17]. Therefore, the dual role of NOD2 in monocyte recruitment and type I IFN production deserves closer attention in relationship with the protective action of MDP against viral infection.

In this report, we define a mechanism for the antiviral activity of MDP against IAV, which requires NOD2-dependent induction of lung CCL2 and type I IFN as well as NOD2- and CCR2-dependent recruitment of Ly6C^high^ monocytes to the lungs. We also demonstrate that the protective effect of MDP treatment necessitates the IPS-1 adaptor molecules involved in IAV recognition to be functional. Furthermore, we show that *N*-glycolyl MDP, a naturally occurring derivative of the classical *N*-aceyl MDP, is more potent at reducing viral burden and that treatment with both forms of MDP restores pulmonary function following IAV infection.

## Results

### MDP treatment depends on NOD2 to improve survival of IAV-infected mice and to reduce pulmonary viral load and inflammation

**Figure 1:**
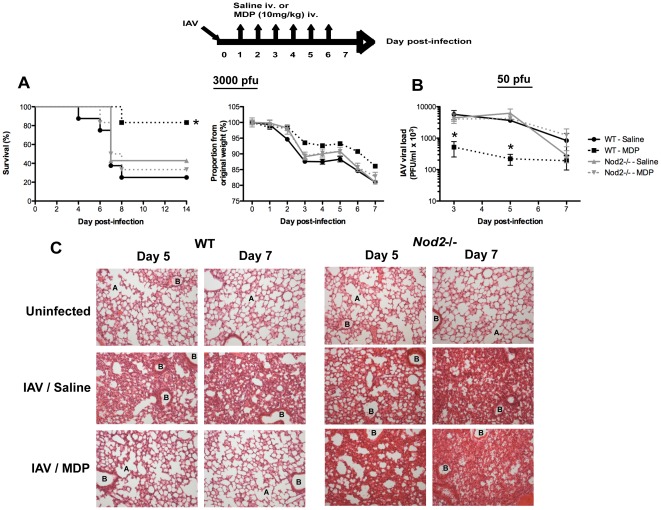
MDP treatment depends on NOD2. Top (in bold): Schematic representation of daily treatment with MDP following IAV infection. (A) WT and *Nod2*-/- mice (n = 10/group) were infected in. with IAV (3000 PFU) and treated daily for 6 days with either saline or MDP (intravenous administration, iv.). Survival (left panel) and body weight (right panel) was monitored daily. Data shown are representative of three independent experiments (*p≤0.05 as compared to saline-treated mice, log-rank test). (B–C) WT and *Nod2*-/- mice (n = 5/group) were infected in. with IAV (50 PFU) and treated as in A. Mice were sacrificed at day 3, 5 and 7 pi. and viral loads were determined from homogenized lungs (B). At day 5 and 7, upper left lobes were harvested and stained with H&E for histological examination by microscopy at 100× magnification (C). A: example of alveolar structure; B: example of bronchiolar structure. Data shown are representative of three independent experiments (*p≤0.05 as compared to saline-treated mice, *t*-test).

MDP and synthetic derivatives have been reported to exhibit antiviral activity against IAV [18]–[20]. In our infection model, daily administration of 10 mg/kg MDP intravenously (iv.) starting at day 1 and ending at day 6 post-infection (pi.) with a sub-lethal dose of IAV was sufficient to significantly reduce viral titers in the lungs of mice (Figure S1). To verify if the protective effect of MDP treatment against IAV depends on NOD2, we first infected wild-type (WT) and *Nod2*-/- mice with a lethal dose of IAV equivalent to ten times the LD50 and treated them with saline or MDP. As shown in Figure 1A, WT animals treated with MDP featured significantly increased survival (*left panel*) and decreased weight loss (*right panel*) when compared to their saline-treated counterpart. Conversely, MDP treatment of *Nod2*-/- animals did not affect survival and body weight loss as compared to saline-treated *Nod2*-/- animals. No significant difference in survival and body weight loss was observed between saline-treated WT and *Nod2*-/- mice with high dose IAV challenge.

In order to better dissect the NOD2-dependent antiviral activity of MDP during the course of IAV infection, WT and *Nod2*-/- mice were infected with a sub-lethal dose of virus and treated or not with MDP as described above. In WT animals, MDP treatment caused a one-log decrease in lung viral loads at day 3 and 5 pi. as compared to saline-treated controls (Figure 1B). No decrease was observed in MDP-treated *Nod2*-/- mice and no significant difference in viral load could be observed between WT and *Nod2*-/- mice treated with saline. Upon histological examination of the lungs, we noticed a marked reduction in leukocyte infiltration as well as decreased airway plugging and alveolar wall thickening in MDP-treated WT mice at day 5 and 7 pi. as compared to saline-treated control animals (Figure 1C). However, the ability of MDP treatment to restore the pulmonary architecture was absent in *Nod2*-/- mice at both day 5 and 7 pi. Collectively, these results show that the protective antiviral activity of MDP against IAV is dependent on NOD2.

### MDP treatment causes NOD2-dependent modulation of lung cytokine production during the course of IAV infection

**Figure 2:**
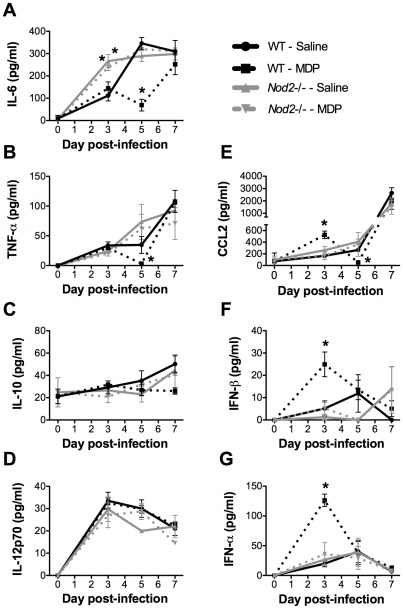
MDP treatment causes NOD2-dependent modulation of pulmonary cytokines. (A–G) WT and *Nod2*-/- mice (n = 5/group) were infected in. with IAV (50 pfu) and treated daily with either saline or MDP (iv.). Prior to infection and at day 3, 5 and 7 pi., levels of various cytokines were determined from homogenized lungs. Data shown are representative of two independent experiments (*p≤0.05 as compared to saline-treated WT mice, *t*-test).

Considering dysregulated immune inflammatory response is the major cause of pathology associated with IAV infection, we next characterized the inflammatory state of the lungs upon MDP treatment during the course of IAV infection. We first measured a panel of inflammatory cytokines directly from lung homogenates of infected WT and *Nod2*-deficient mice treated or not with MDP. In MDP-treated WT mice, levels of IL-6 and TNF-α were significantly reduced at day 5 pi. (Figure 2A–B). The rise in both IL-6 and TNF-α production between day 5 and day 7 in WT mice treated with MDP to a similar level than saline-treated animals suggests that these cytokines are required for the control and/or resolution of IAV infection but that a delay in their production could be beneficial to the host. Indeed, it has recently been shown that both IL-6 and TNF-α were essential in the late phase of IAV infection by promoting neutrophil survival and viral clearance [21] as well as by down-modulating the extent of lung immunopathology [22] respectively. Interestingly, *Nod2*-/- mice showed an early and significant increase in lung IL-6 but not TNF-α as compared to saline-treated WT animals at day 3 pi. irrespective of treatment, a finding that was recapitulated during *in vitro* alveolar macrophage infection (Figure 2A–B and Figure S2A–B). Despite its low level of secretion, IL-10 tended to be reduced at days 5 and 7 pi. in WT mice treated with MDP relative to untreated WT mice consistent with the documented positive correlation between this cytokine and viral load [23] (Figure 2C). No significant difference in the production of IL-12p70 (Figure 2D) was detected in lungs of MDP-treated WT mice as compared to saline-treated animals during the course of the infection. Interestingly, CCL2 levels were significantly increased at day 3 pi. in the lungs of WT animals treated with MDP as compared to saline-treated controls (Figure 2E). Such increase observed at day 3 in MDP-treated relative to saline-treated mice was transient since CCL2 production was significantly lower in mice administered with MDP at day 5 pi. before rising again at day 7. Importantly, the modulatory effect of MDP treatment on lung cytokine production during IAV infection was dependent on NOD2 since no significant difference in levels of all measured cytokines could be observed between MDP-treated and saline-treated *Nod2*-deficient mice (Figure 2A–E).

Type I IFNs are critical for the control of IAV infection [13], [14] and IAV is known to suppress their production during the early phase of infection [24]. On the other hand, MDP is known to cause NOD2-dependent induction of type I IFNs via RIP2 and IRF5 [12]. We therefore measured the levels of type I IFNs in lung homogenates of IAV-infected WT and *Nod2*-deficient mice treated or not with MDP. Consistent with published findings [15], IFN-β tended to be reduced in the lungs of infected *Nod2*-/- mice as compared to WT animals during the early phase of infection and *Nod2*-/- alveolar macrophages produced significantly less IFN-β than WT cells in response to IAV (Figure 2F and Figure S2C). In sharp contrast to inflammatory cytokines (IL-6, TNF-α), MDP treatment of WT mice significantly increased both IFN-β and IFN-α production in the lungs as compared to saline-treated mice at day 3 pi. (Figure 2F–G). Again, the effect of MDP treatment was NOD2-dependent since there was no difference in induction of type I IFN in MDP-treated *Nod2*-deficient animals during the course of infection. In recall experiments, total lung cells derived from infected WT mice treated with MDP and re-challenged *ex vivo* with virulent IAV also produced increased type I IFN and inhibited IAV replication in a NOD2-dependent manner (Figure S3). Altogether, these findings show that during the course of IAV infection, MDP treatment modulates the production of inflammatory cytokines in a NOD2-dependent fashion and causes an early and NOD2-dependent increase in CCL2 and type I IFNs.

### MDP treatment causes a NOD2-dependent increase in the pool of circulating monocytes

**Figure 3:**
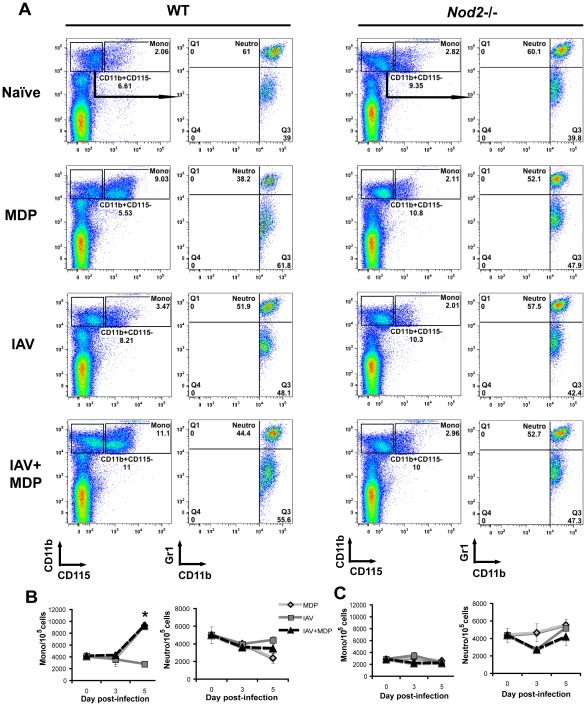
MDP treatment enhances the number of blood monocytes. (A–C) WT and *Nod2*-/- mice (n = 3/group) were infected or not in. with IAV (50 pfu) and treated daily with either saline or MDP (iv.). At day 5 pi., blood cells were subjected to flow cytometry (A). Mono: monocytes; Neutro: neutrophils. Numbers indicate cell population percentages within gates. Relative number of monocytes and neutrophils were determined in naïve mice and at day 3 and 5 pi. (B and C). Data shown are representative of two independent experiments (*p≤0.05 as compared to IAV-infected and saline-treated mice, *t*-test).

Monocytes are a multipotent subset of bone marrow-derived cells that can migrate to infected tissues and further differentiate into specialized types of macrophages and dendritic cells [25]. MDP has previously been shown to stimulate hematopoiesis when administered systemically [6]. In addition, the secretion of CCL2 and type I IFNs, which have well-characterized functions in monocyte recruitment [25], was enhanced in the lungs of IAV-infected and MDP-treated mice in a NOD2-dependent fashion (Figure 2E–G). Therefore, we investigated the effect of treatment on the number of circulating monocytes as well as neutrophils. Blood was collected from WT and *Nod2*-deficient mice infected or not with IAV and treated or not with MDP and cells were stained with Abs against CD11b, CD115 and Gr1 to identify monocytes (CD11b^+^CD115^+^) and neutrophils (CD11b^+^CD115^−^Gr1^+^). Figure 3A shows representative dot plots obtained at day 5 pi. and results are quantified in Figures 3B and 3C. In infected and uninfected WT animals treated with MDP, the number of circulating monocytes was significantly enhanced at day 5 pi. as compared to infected animals treated with saline (Figure 3B, *left panel*). In contrast, although MDP treatment tended to decrease the number of blood neutrophils at day 5 pi., no significant difference could be observed between infected and uninfected mice treated with MDP or between infected mice treated or not with MDP (Figure 3B, *right panel*). The effect of MDP was NOD2-dependent since no significant difference was observed between all groups in the number of blood monocytes and neutrophils in *Nod2*-deficient mice (Figure 3C). Therefore, systemic administration of MDP causes a selective increase in circulating monocytes that is dependent on NOD2 and independent on IAV infection.

### MDP treatment causes NOD2-dependent transient increase in recruitment of Ly6C^high^ monocytes to the lungs of IAV-infected mice

**Figure 4:**
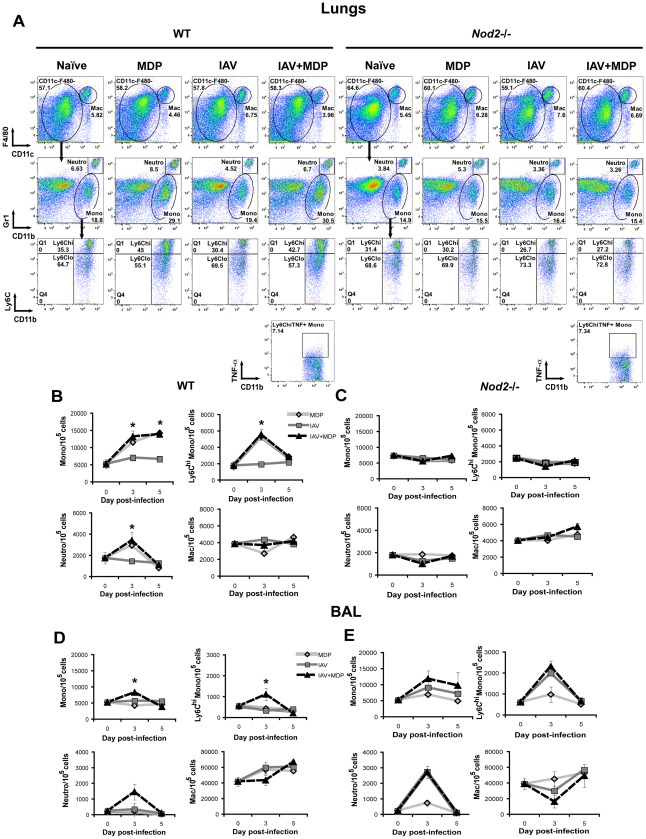
MDP treatment causes NOD2-dependent recruitment of Ly6C^high^ monocytes to the lungs. (A–E) WT and *Nod2*-/- mice (n = 3/group) were infected or not in. with IAV (50 pfu) and treated daily with either saline or MDP (iv.). At day 3 pi., total lung cells were subjected to flow cytometry (A). Mac: macrophages; Mono: monocytes; N: neutrophils. Numbers indicate cell population percentages within gates. Relative number of monocytes, Ly6C^high^ monocytes, neutrophils and macrophages were determined in the lungs (B–C) and BAL (D–E) in mice and at day 3 and 5 pi. Data shown are representative of three independent experiments (*p≤0.05 as compared to IAV-infected and saline-treated mice, *t*-test).

A recent report by Seo and colleagues suggests that the protective effect of type I IFNs during IAV infection is linked to their role in triggering the preferential recruitment of Ly6C^high^ monocytes via MCP-1 (also known as CCL2) over neutrophils to the lungs [17]. Since we observed a NOD2-dependent increase in CCL2, type I IFNs and circulating monocytes following MDP treatment of IAV-infected mice (Figure 2E–G and Figure 3), we investigated whether such treatment affected populations of monocytes, neutrophils and macrophages in the lungs during the course of infection. To do so, total lung cells and bronchoalveolar lavage (BAL) cells from WT and *Nod2*-/- mice infected or not with IAV and treated or not with MDP were isolated and stained with Abs against F4/80, CD11c, CD11b, Gr1 and Ly6C. Cells were then gated by flow cytometry to identify macrophages (F4/80^high^CD11c^high^), monocytes (CD11b^+^Gr1^int^) and neutrophils (CD11b^+^Gr1^high^). To identify inflammatory monocyte subsets, monocyte population was gated back with CD11b^+^Ly6C^high^ and this cell population was further analyzed for TNF-α secretion in infected and treated WT and *Nod2*-/- mice. Figure 4A and Figure S4 serve as a representative examples and quantification is depicted in Figures 4B–C (lungs) and 4D–E (BAL). In WT mice infected or not with IAV and treated with MDP, the relative number of monocytes in the lungs was significantly enhanced at day 3 and 5 pi. when compared to IAV-infected mice treated with saline (Figure 4B, *top left panel*). MDP treatment of infected and uninfected mice also induced a transient and significant increase in the relative number of Ly6C^hi^ monocytes as compared to saline-treated IAV-infected mice at day 3 pi. (Figure 4B, *top right panel*). This increase in proportion of Ly6C^high^ monocytes in MDP-treated mice was accompanied by a transient increase in the proportion of neutrophils (Figure 4B, *bottom left panel*) while the macrophage fraction did not vary significantly between MDP-treated groups and between IAV-infected groups (Figure 4B, *bottom right panel*). In *Nod2*-deficient animals, MDP treatment did not cause any difference in the relative number of lung monocytes, Ly6C^high^ monocytes, neutrophils and macrophages during the course of infection (Figure 4C). Consistent with their inflammatory phenotype, Ly6C^high^ monocytes from both WT and *Nod2*-/- mice infected with IAV and treated with MDP secreted TNF-α (Figure 4A, *bottom*).

In the BAL, monocytes were increased at day 3 pi. in IAV-infected WT animals treated with MDP as compared to those treated with saline (Figure 4D, *top left panel* and Figure S4). Similar to what was observed in the lungs, relative numbers of Ly6C^high^ monocytes (Figure 4D, *top right panel*) and neutrophils (Figure 4D, *bottom left panel*) were transiently increased in the BAL of IAV-infected and MDP-treated WT mice at day 3 pi. although neutrophils did not reach statistical significance. Alveolar macrophages remained unaffected by MDP treatment in the BAL of IAV-infected mice (Figure 4D, *bottom right panel*). Interestingly, while MDP treatment had similar effect in infected and uninfected WT mice with respect to the modulation of cellular populations in the blood and lung tissue, MDP alone did not cause any change in the relative number of all cell types investigated in the BAL (Figure 4D). In *Nod2*-deficient mice, MDP treatment did not have any significant effect on the relative number of monocytes, Ly6C^high^ monocytes, neutrophils and macrophages either when comparing IAV-infected groups treated or not with MDP or when comparing mice treated with MDP alone at day 3 and 5 to baseline (day 0) (Figure 4E). Therefore, MDP treatment causes a transient NOD2-dependent increase in pulmonary Ly6C^high^ monocytes and, to a lesser extent, neutrophils. This increase occurs in lung tissue in the presence or absence of IAV infection but occurs in the airways only in IAV-infected mice.

### The protective activity of MDP treatment against IAV depends on CCR2 and IPS-1

**Figure 5:**
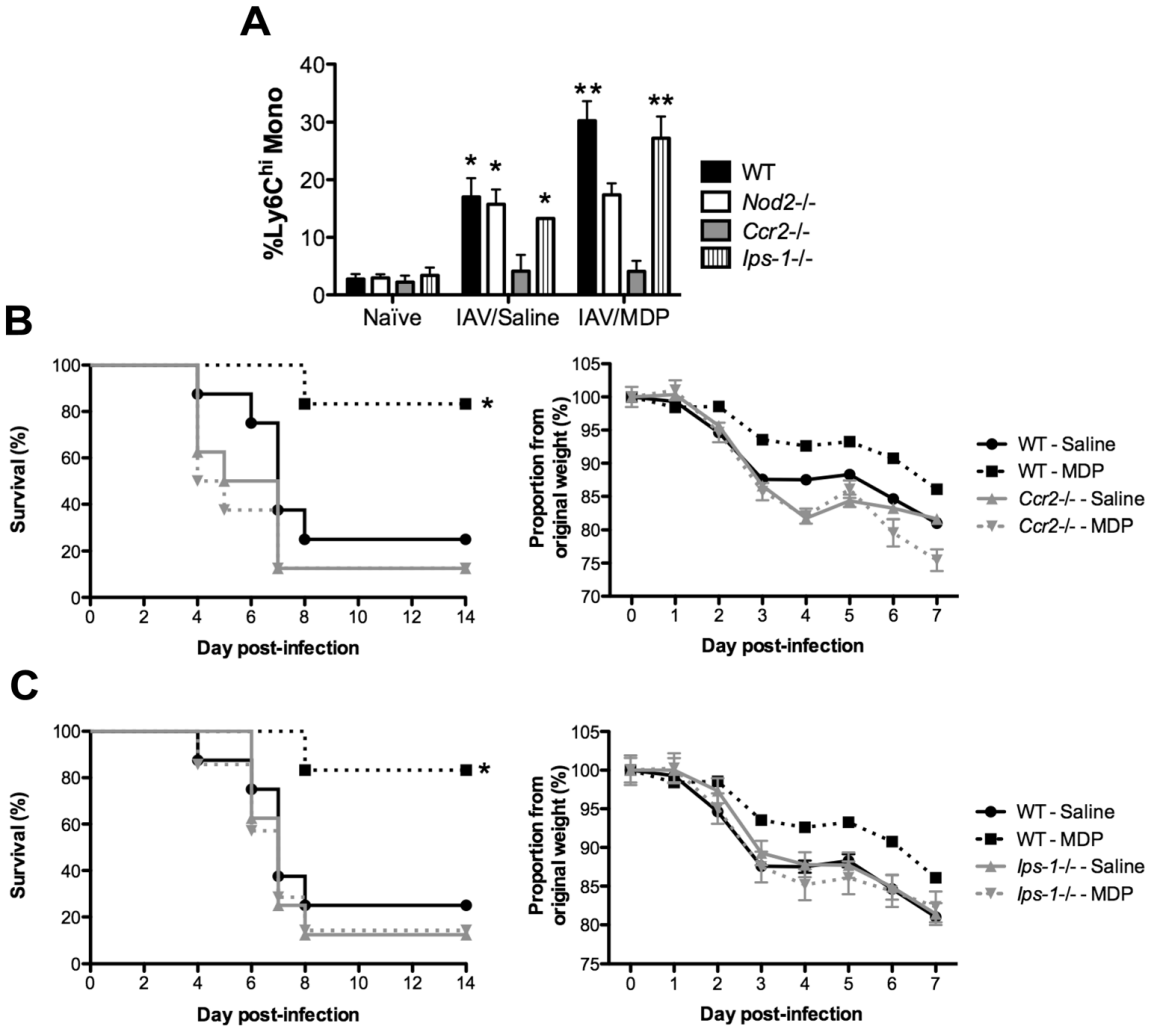
MDP treatment requires CCR2 and IPS-1. (A) WT, *Nod-2-/-, Ccr2*-/- and *Ips-1*-/- mice (n = 3/group) were infected in. with IAV (50 pfu) and treated daily with either saline or MDP (iv.). Frequency of Ly6C^high^ monocytes was determined in the BAL of naïve mice and in infected mice at day 3 pi. Data shown are representative of two independent experiments (* p≤0.05 as compared to naïve mice of the same genotype, **p≤0.05 as compared to saline-treated mice of the same genotype, *t*-test). (B and C) Survival (left panels) and body weight (right panels) was monitored daily in WT, *Ccr2-/-* and *Ips-1*-/- mice (n = 10/group) infected with a lethal dose of virus (3000 pfu) and treated as in A. Data shown are representative of two independent experiments (*p≤0.05 as compared to saline-treated mice, log-rank test).

Migration of Ly6C^high^ monocytes from the bone marrow occurs when CCL2 binds to its receptor CCR2 expressed at high levels on these cells [26]. To ask whether CCR2 is required for the protective effect of MDP-treatment in IAV-infected mice, *Ccr2*-/- animals were infected with a sub-lethal or a lethal dose of virus and treated or not with MDP. As depicted in Figure 5A, *Ccr2*-deficient mice infected with a sub-lethal dose of virus and treated with saline failed to increase their frequency of Ly6C^high^ monocytes into the lungs. In addition, MDP-treatment of these mice was unable to cause an increase in the percentage of Ly6C^high^ monocytes as compared to saline-treated controls at day 3 pi., unlike what was observed in WT animals. Importantly, the protective effect of MDP treatment upon infection with a lethal dose of virus was CCR2-dependent since no difference in survival (Figure 5B, *left panel*) and in body weight loss (Figure 5B, *right panel*) was observed between saline- and MDP-treated *Ccr2*-deficient mice. Thus, the protective effect of MDP treatment against IAV is linked to the NOD2- and CCR2-dependent recruitment of Ly6C^high^ monocytes to the lungs.

NOD2-mediated sensing of MDP is known to occur via RIP2, independently of the IPS-1 adaptor molecule. However, IPS-1 acts downstream of the RIG-I PRR implicated in IAV viral RNA recognition [27]. To investigate whether MDP treatment could have a protective effect on IAV-infected mice with compromised innate response to the virus, *Ips*-*1*-/- animals were infected with IAV and treated or not with MDP. Due to the importance of Ly6C^high^ monocytes recruitment for the protective effect of MDP treatment, we first verified if these cells were recruited following treatment of knockout mice. *Ips-1*-deficient animals infected with a sub-lethal dose of virus and treated with MDP all showed an increased proportion of Ly6C^high^ monocytes in the lungs as compared to uninfected mice and a further increase upon MDP treatment as compared to saline-treated animals at day 3 pi., similar to WT mice (Figure 5A). Upon lethal challenge with IAV, *Ips-1*-/- mice treated with MDP did not show any significant difference in survival (Figure 5C, *left panel*) and weight loss (Figure 5C, *right panel*) as compared to those treated with saline. Thus, despite MDP treatment and Ly6C^high^ monocyte recruitment, the immunomodulating activity of MDP during IAV infection requires functional innate host response via IPS-1.

### *N*-glycolyl MDP is more potent than *N*-acetyl MDP and restores lung function in IAV-infected mice

**Figure 6:**
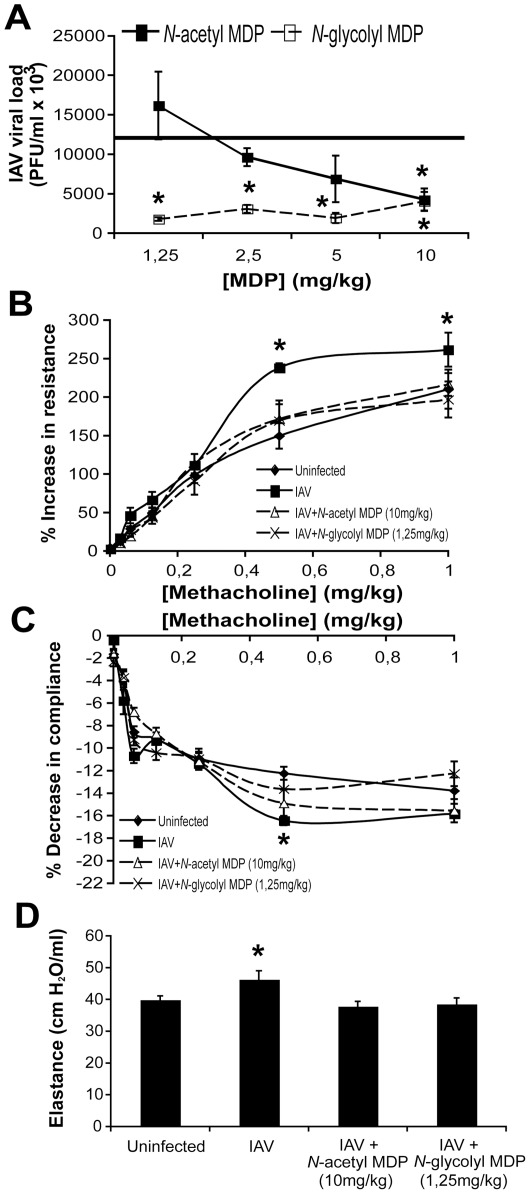
*N*-glycolyl MDP is more potent than *N*-acetyl MDP. (A) WT mice (n = 5/group) were infected in. with IAV (50 pfu) and treated daily with saline or various doses of *N*-acetyl MDP or *N*-glycolyl MDP (iv.). Mice were sacrificed at day 3 pi. and viral loads were determined from homogenized lungs. The line represents IAV viral load in the lungs of saline-treated mice. (*p≤0.05 as compared to saline-treated mice, *t*-test). (B–D) WT mice (n = 8/group) were infected or not in. with IAV (50 pfu). From day 1 to day 4 pi., infected mice were treated daily with either saline, *N*-acetyl MDP (10 mg/kg) or *N*-glycolyl MDP (1.25 mg/kg) iv. At day 5 pi., airway resistance (B) and compliance (C) were both measured upon methacholine challenge and baseline elastance (D) was measured prior to methacholine administration using the flexiVent apparatus. (* *p*≤0.05 as compared to uninfected mice, *t*-test).

The conversion of the *N*-acetyl group at carbon 2 of the muramic acid moiety of MDP to a *N*-glycolyl group by mycobacteria and related organisms has recently been demonstrated to render the molecule more potent at activating NOD2- and RIP2-dependent responses [12], [28]. To ask whether this modification would also improve the potency of MDP as a treatment against IAV, WT mice were infected and treated with various concentrations of both forms of MDP. As shown in Figure 6A, 10 mg/kg *N*-acetyl MDP, the form and dose of MDP used throughout this study, caused a significant reduction in IAV viral load in the lungs as compared to saline-treated mice (horizontal line, 11839±1234 PFU/ml ×10^3^). At the same 10 mg/kg concentration, *N*-glycolyl MDP caused a similar decrease in viral load than *N*-acetyl MDP. However, upon decreasing the administered concentration, *N*-acetyl MDP could no longer significantly reduce lung viral load while *N*-glycolyl MDP remained active down to a concentration of 1.25 mg/kg. To characterize the physiological impact of treatment with both forms of MDP during IAV infection, we analyzed lung function in IAV-infected mice treated or not with active concentrations of *N*-acetyl MDP (10 mg/kg) or *N*-glycolyl MDP (1.25 mg/kg). At day 5 pi., animals were tracheotomized and ventilated using the flexiVent apparatus in order to evaluate airway responsiveness to methacholine challenge and baseline elastance. Airway resistance and pulmonary compliance to methacholine were respectively significantly enhanced and decreased in saline-treated IAV-infected mice as compared to uninfected animals (Figure 6B and 6C). In contrast, both parameters did not vary significantly from uninfected mice in IAV-infected mice treated with active concentrations of either form of MDP. In line with these results, pulmonary elastance (reciprocal of compliance) measured prior to methacholine challenge was significantly enhanced in saline-treated IAV-infected mice as compared uninfected mice while no difference was observed in MDP-treated mice (Figure 6D). Taken together, these results show that the *N*-glycolyl MDP derivative is more potent than the classical MDP and that the antiviral activity of MDP against IAV correlates with improved lung function.

## Discussion

Following the discovery of NOD2 as a major susceptibility gene for Crohn's disease and as the host sensor for bacterial-derived MDP, its role in promoting antiviral immunity has not surprisingly been neglected despite the long-standing observation that MDP and derivatives could provide protection against viral infection. The first direct link between NOD2 and antiviral immunity has recently been provided by Sabbah and colleagues, who have identified a role for NOD2 in detection of viral ssRNA [15]. Although most *in vivo* studies performed by this group were done using respiratory syncytial virus, the authors conclude that NOD2 acts as a general activator of antiviral immunity against ssRNA viruses. While we did observed NOD2-dependent IFN-β secretion by macrophages in response to IAV, a ssRNA virus (Figure S2), we could not find any major role for NOD2 itself (independently of MDP treatment) in protection against IAV using a sub-lethal or a lethal dose of virus (Figure 1A–B). Instead, we found that NOD2 was the host PRR responsible for the antiviral activity of MDP and provided further explanations on the mechanism underlying this effect.

MDP treatment modulated cytokine secretion in the lungs of IAV-infected mice. The effect of treatment was transient, causing an early decrease in IL-6 and TNF-α as well as an increase in type I IFN. The net result of this altered cytokine balance might have been reduced inflammation associated with excessive early IL-6 and TNF-α secretion and increased viral clearance by overcoming the NS1-mediated inhibitory effect of the virus on type I IFN production [24]. Later increase in IL-6 and TNF-α secretion in MDP-treated mice up to a similar level than saline-treated animals might also have contributed to protection since IL-6 is known to promote neutrophil survival and function in the late phase of infection while TNF-α deficiency has recently been demonstrated to exacerbate immunopathology in a mouse model of acute IAV infection [21], [22]. While MDP has been shown to induce type I IFN via IRF5 [12], the mechanism by which MDP treatment augmented type I IFN secretion in the lungs remains unclear. The contribution of IRF5 as well as IRF3 and IRF7 activation by IAV during infection and MDP treatment is the subject of ongoing investigation.

A recent human study has shown that IAV is capable of infecting monocytes irrespective of their cell surface phenotype and this cell type was more resistant than differentiated macrophages to the virus [29]. In our study, lung CCL2 levels and circulating blood monocytes were increased in IAV-infected WT mice treated with MDP. Within the pulmonary environment, we also observed a NOD2-dependent relative increase in monocytes, more specifically in the Ly6C^high^ monocyte subset, in both lung tissue and BAL of IAV-infected mice treated with MDP as opposed to those treated with saline. Ly6C^high^ monocytes recruitment was dependent on CCR2 and MDP treatment was ineffective in animals deficient in this receptor. The role of Ly6C^high^ monocytes during IAV infection is controversial. *Ccr2*-deficiency has been associated with decreased immunopathology and mortality due to impaired macrophages recruitment into the lungs of IAV-infected mice [30]. However, the ability of *Ccr2*-/- mice to clear IAV was also shown to be delayed due to a blockage in recruitment of tipDCs to the lungs and decreased priming of viral antigen-specific CD8 T cells [31]. In addition, type I IFN signaling during IAV infection has been demonstrated to positively regulate the influx of Ly6C^high^ monocytes into the lungs [17]. Therefore, while excessive recruitment of Ly6C^high^ monocytes might be detrimental during pulmonary IAV infection, an early and transient increase relative to other innate effector cells types such as what we observed upon MDP treatment could be beneficial to enhance both viral clearance and priming of antigen-specific CD8 T cell response leading to a faster resolution of infection.

The control of adaptive immune responses by NOD2 is a subject under intense investigation [32]. While systemic NOD2 stimulation by MDP alone as been demonstrated to induce a T helper 2 (Th2) type of adaptive response, the generation of a Th1 response following co-stimulation with MDP and Toll-like receptor (TLR) agonists was NOD2-dependent [33]. Treatment with bacteria-derived MDP during a viral infection such as IAV, which itself activates an array of innate immune receptors including various TLRs, may therefore be expected to enhance IAV-specific Th1 type immunity and potentially anti-IAV antibody titers. Indeed, NOD2-dependent antibody production has recently been reported in the context of streptococcal infection [34]. Efforts are currently underway to evaluate the impact of MDP treatment and of Ly6C^high^ monocytes recruitment on the unfolding of the adaptive response in the lungs during IAV infection and in the context of vaccination against the virus.

The ability of exogenous MDP to induce a NOD2-dependent, transient increase in levels of various cytokines in the serum when administered systemically has been well studied [33]. In addition, we have shown here that MDP treatment alone in the absence of IAV infection enhanced the number of circulating monocytes, again via NOD2. Despite specific activation of the NOD2 PRR, the protective action of immunomodulators such as MDP and derivatives is not specific toward the pathogen per se and is often described as a “boost of natural immunity”. Clear evidence for such an affirmation comes from the observation that mice deficient in IPS-1, a key adaptor molecule in innate immunity to IAV [27], cannot be protected by MDP treatment despite Ly6C^high^ monocytes recruitment to the lungs. Hence, efforts are being made in order to generate immunomodulatory compounds that conserve protective “boosting” activity with minimal toxicity. Examples include Murabutide, MDP-Lys (L18) (also known as Romurtide) and L-MTP-PE (also known as Mifamurtide), three MDP derivatives with low toxicity, which are promising for the treatment of various infections and cancers such as HIV and osteosarcoma [6], [7], [35]–[38]. In this study, we have shown that the natural MDP derivative *N*-glycolyl MDP has a more potent antiviral activity against IAV than the classical *N*-acetyl MDP, consistent with the finding that *N*-glycolylation of MDP increases its NOD2-stimulating activity [28]. Ongoing studies are aimed at evaluating the toxicity of low dose *N*-glycolyl MDP and at establishing whether it may have protective activity in different models of viral infection and at different times following infection.

To conclude, the determination of key cellular and molecular events induced by MDP treatment is expected to lead to a better understanding of both the correlates of a protective response against IAV and the mechanism of action of muramyl peptide immunomodulators. In parallel, further investigation on the use of clinically approved and novel MDP analogues for the immunotherapy of IAV infection should be considered.

## Materials and Methods

### Ethics statement

This study was carried out in strict accordance with the recommendations in the Guide for the Care and Use of Laboratory Animals of the Canadian Council on Animal Care (CCAC). The protocol was approved by the Committee on the Ethics of Animal Experiments of Université Laval (Permit Number: 09-121-2).

### Mice

WT C57Bl/6 and *Nod2*-deficient mice were purchased from the Jackson Laboratory (Bar Harbor, Maine, USA). *Ccr2*-deficient mice were kindly provided by Dr. Serge Rivest (CHUQ Research Center, Université Laval, Quebec, Canada). *Ips-1*-deficient mice were kindly provided by Dr. Shizuo Akira (Osaka University, Osaka, Japan). *Nod2*-/-, *Ccr2*-/- and *Ips-1*-/- breeding colonies were established at the CHUQ Research Center. All study mice were 4 to 6 weeks old and experiments were conducted in accordance with the guidelines of animal research ethics boards of Laval University.

### Virus, cells and reagents

All infections were performed with Influenza virus strain A/Puerto Rico/8/34 (H1N1). IAV was propagated and isolated from Madin-Darby canine kidney (MDCK) cells and titrated using standard plaque assay in MDCK cells [39]. The MDCK cell line, alveolar macrophages and total lung cells (10^5^ cells/well, 96-well plate) were cultured in MEM supplemented with 10% heat-inactivated FBS and gentamycin sulfate (40 µg/ml). *N*-acetyl MDP and *N*-glycolyl MDP were purchased from Invivogen (San Diego, CA, USA) and reconstituted with endotoxin-free water (Invivogen).

### Virus infection of mice and treatment

For *in vivo* protocols, as summarized in Figure 1 (top panel), mice were infected in. at day 0 with 50 PFU of IAV except for survival experiments where mice were infected with 3000 PFU of the virus. Animals were randomized at day 1 and then treated iv. with saline (NaCl 0.9%) or with reconstituted MDP dissolved in saline at the appropriate concentration (1.25–15 mg/kg) daily from day 1 up to day 6 pi. For survival experiments (3000 PFU IAV), animals were monitored daily for health status and weight determination for up to 14 days post-infection. For all other experiments (50 PFU IAV), animals were sacrificed at day 3, 5 or 7 pi. and lungs were harvested and homogenized for viral load determination by standard plaque assay in MDCK cells.

### Cyokine measurement

IL-6, TNF-α, IL-10, IL-12p70 and CCL2 concentrations were determined directly from lung homogenates (expressed as pg/ml of homogenized lungs, homogenized in 1 ml) or cell culture supernatant using the BD Cytometric Bead Array (CBA) mouse inflammatory kit (BD Biosciences, San Diego, CA, USA) and analyzed using BD FACSArray Bioanalyzer System (BD Biosciences, Ontario, Canada). Concentrations of IFN-α and IFN-β were determined by ELISA (PBL InterferonSource, Piscataway, NJ, USA).

### Flow cytometry

Single-cell suspensions obtained from blood, collagenase- and DNase-treated lungs or BALs were first incubated with anti-CD16/32 (BioLegend, San Diego, CA, USA) to block non-specific Ab interaction with Fc receptors. Then, cells were stained with V500-conjugated anti-CD45 (BD Biosciences, San Diego, CA, USA) as an appropriate gating strategy to visualize granulocytes and mononuclear cell populations, with APC-conjugated anti-CD115 (BioLegend, San Diego, CA, USA), V450-conjugated anti-Gr1 (Ly6C/Ly6G) (BD Biosciences, San Diego, CA, USA), PE-Cy7-conjugated anti-F4/80 (BioLegend, San Diego, CA, USA), APC-Cy7-conjugated anti-CD11b and FITC-conjugated anti-CD11c (BD Biosciences, San Diego, CA, USA). For intracellular cytokine staining, cells were treated with GolgiPlug (BD Biosciences, San Diego, CA, USA) for 5 h. Cells were washed and blocked with anti CD16/32 followed by staining with appropriate Abs against cell surface markers. Cells were then washed, permeabilized, and stained according to the manufacturer's instructions with FITC-conjugated anti-TNF-α (BD Biosciences, San Diego, CA, USA). Flow cytometry was performed using BD LSR II (BD Biosciences, Ontario, Canada) with FACSDiva software Version 6.1.2 (BD Biosciences, Ontario, Canada) on 100,000 cells per sample. Analysis was performed using FlowJo Software (Tree Star Inc., Ashland, OR, USA).

### Analysis of pulmonary function

Mice were anaesthetized with ketamine-xylazine (100 mg/kg ketamine, 10 mg/kg xylazine), tracheotomized and connected to a small animal ventilator (flexiVent, SCIREQ, Inc., Montreal, Quebec, Canada). Then, animals were paralyzed with pancuronium (0.033 mg/kg) and canulated at the level of the jugular vein. Mice were ventilated with a tidal volume of 10 ml/kg, inspiratory/expiratory ratio of 66.67%, respiratory rate of 150/min. Positive end-expiratory pressures (PEEP) were maintained by submerging the expiratory limb from the ventilator into a water trap (2.5–3 cm H_2_O pressure). Increasing concentrations of methacholine (0.03–1 mg/kg) were injected iv. via the jugular vein. Baseline resistance was restored before each subsequent methacholine administration. The flexiVent apparatus ventilates the mouse and then generates pressure–volume curves over 15 sec. intervals where a snapshot measured is obtained before continuing the respirations. The flexiVent is calibrated for gas compressibility and resistive and accelerative losses in all the connections and calculates resistance, compliance and elastance on the basis of the entered mouse weight using the flexiVent 5.1 software.

### Statistical analysis

Student's *t* tests (two-tailed, unpaired) were performed except for survival experiments where data were analyzed by the log-rank test using the XLSTAT software (Addinsoft). Differences were considered significant at *p*≤0.05.

### Online supplemental material

Figure S1 demonstrates the MDP dose-response of mice infected with IAV. Figure S2 compares WT and *Nod2*-/- alveolar macrophages treated with MDP or infected with IAV. Figure S3 shows results from *ex vivo* recall experiments performed with WT and *Nod2*-/- mice. Figure S4 depicts the flow cytometry analysis of cellular recruitment in the BAL of WT and *Nod2*-/- mice infected or not with IAV and treated or not with MDP.

## Supporting Information

Figure S1 WT mice (n = 5/group) were infected in. with IAV (50 PFU) and treated daily for 6 days with either saline or indicated doses of MDP (iv.). Mice were sacrificed at day 3, 5 and 7 pi. and viral loads were determined from homogenized lungs. Data shown are representative of two independent experiments.
(TIF)

Figure S2 Alveolar macrophages from naïve WT and *Nod2*-/- mice were left untreated or were stimulated with MDP (10 µg/ml) or IAV (1 MOI) for 24 h (A–C). IL-6, TNF-α and IFN-β levels were determined from cell culture supernatants. Data shown are representative of two independent experiments (*p≤0.05 as compared to WT cells under the same condition, *t*-test).
(TIF)

Figure S3 (A–B) WT and *Nod2*-/- mice (n = 3/group) were infected and treated daily with either saline or MDP (iv.). At day 3 pi., total lung cells from infected and treated WT and *Nod2*-/- mice were cultured *ex vivo*. Adherent cells were re-infected with IAV (1 MOI) for 16 hours. IFN-β and IFN-α (A) levels as well as viral loads (B) were determined from cell culture supernatants. Data shown are representative of two independent experiments (*p≤0.05 as compared to saline-treated mice, *t*-test).
(TIF)

Figure S4 WT and *Nod2*-/- mice (n = 3/group) were infected or not with IAV and treated daily with either saline or MDP (iv.). At day 3 pi., BAL cells were subjected to flow cytometry analysis. Mac: macrophages; Mono: monocytes; N: neutrophils. Numbers indicate cell population percentages within gates. Data shown are representative of three independent experiments.
(TIF)
