# Supplementary figures and images for: Muramyl Dipeptide Induces NOD2-Dependent Ly6Chigh Monocyte Recruitment to the Lungs and Protects Against Influenza Virus Infection
Source: PLoS One. 2012 May 9;7(5):e36734. doi: 10.1371/journal.pone.0036734 (PMC3348889; doi:10.1371/journal.pone.0036734)

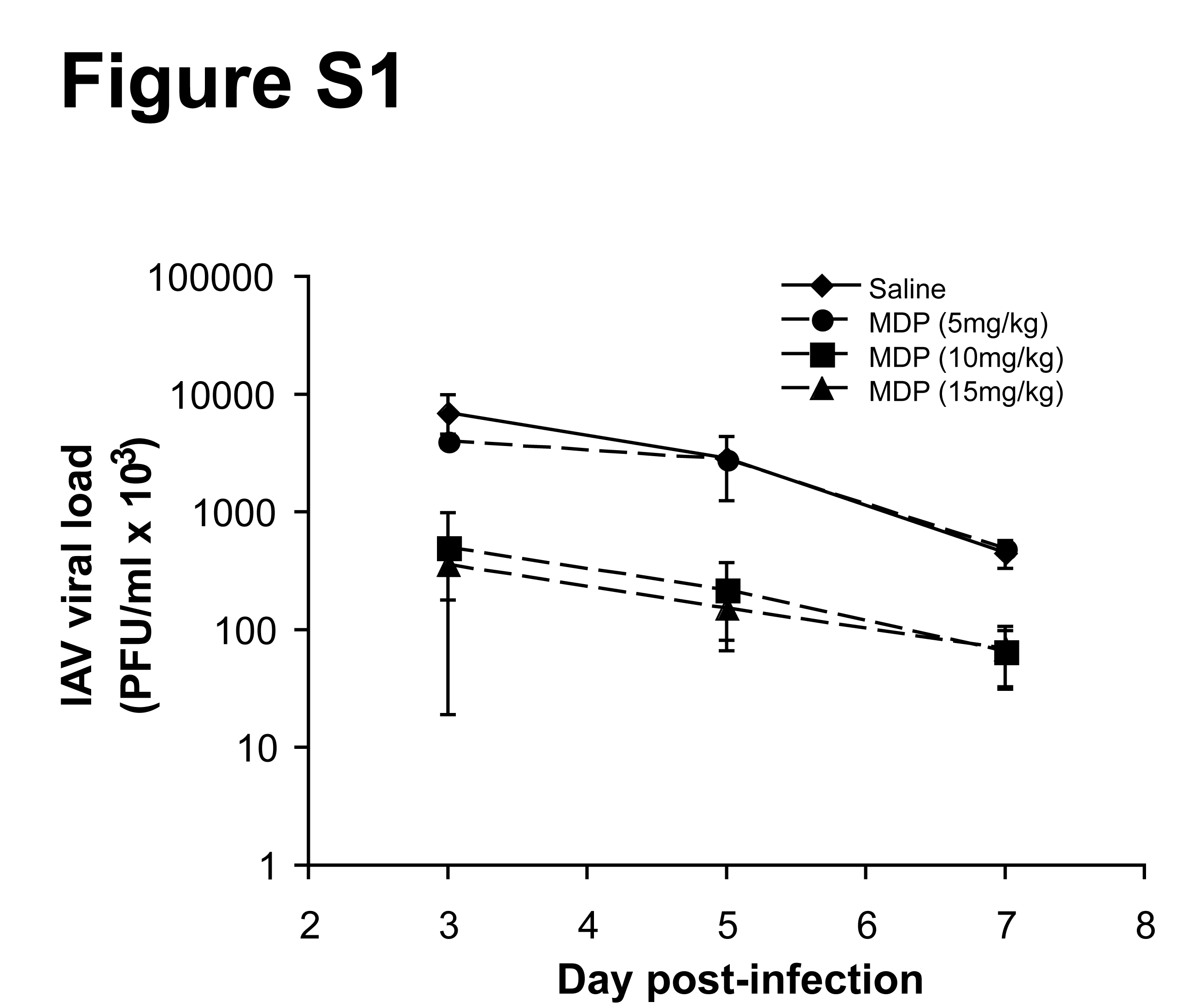

Supplement: Figure S1 — WT mice (n = 5/group) were infected in. with IAV (50 PFU) and treated daily for 6 days with either saline or indicated doses of MDP (iv.). Mice were sacrificed at day 3, 5 and 7 pi. and viral loads were determined from homogenized lungs. Data shown are representative of two independent experiments. (TIF) [file pone.0036734.s001.tif]

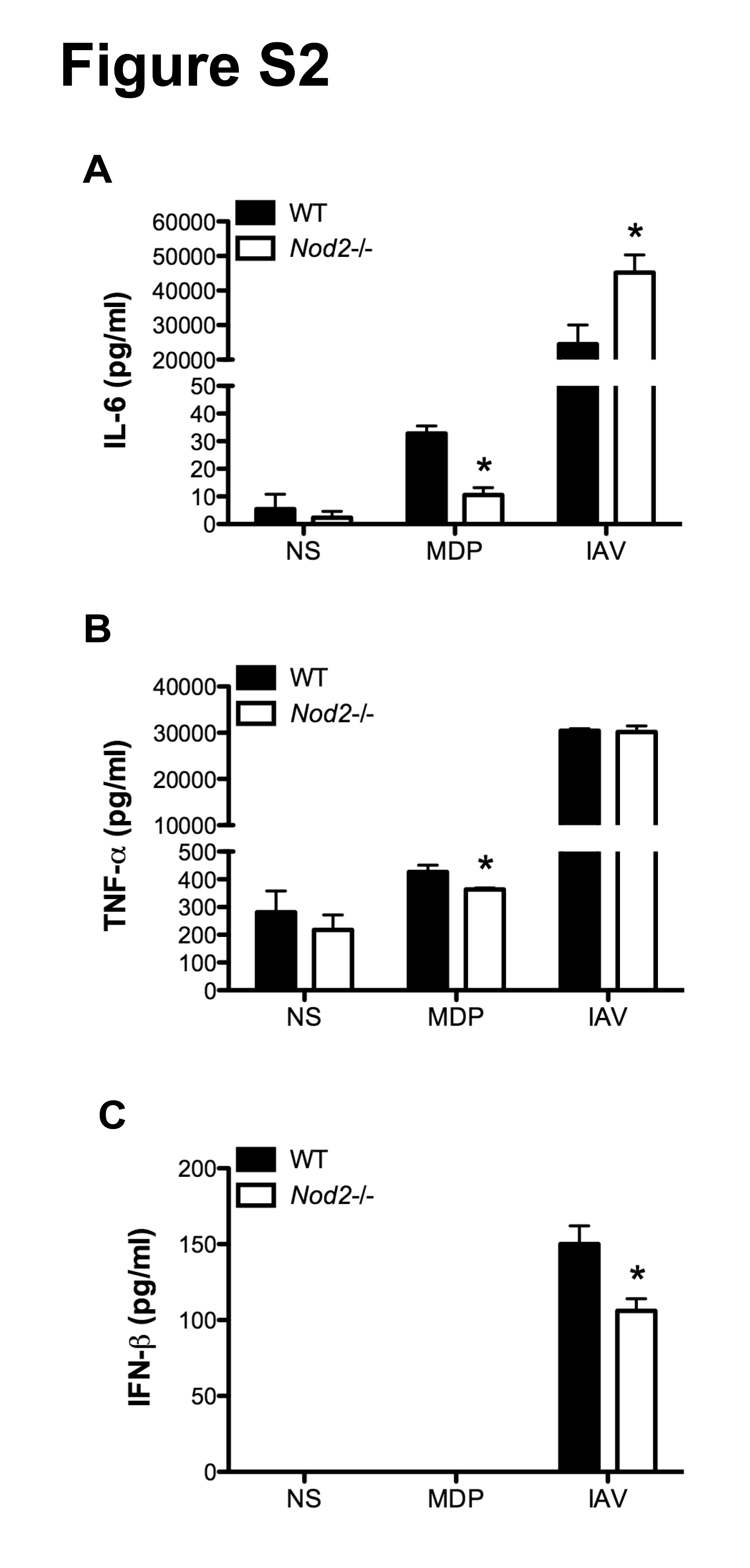

Supplement: Figure S2 — Alveolar macrophages from naïve WT and Nod2-/- mice were left untreated or were stimulated with MDP (10 µg/ml) or IAV (1 MOI) for 24 h (A–C). IL-6, TNF-α and IFN-β levels were determined from cell culture supernatants. Data shown are representative of two independent experiments (*p≤0.05 as compared to WT cells under the same condition, t-test). (TIF) [file pone.0036734.s002.tif]

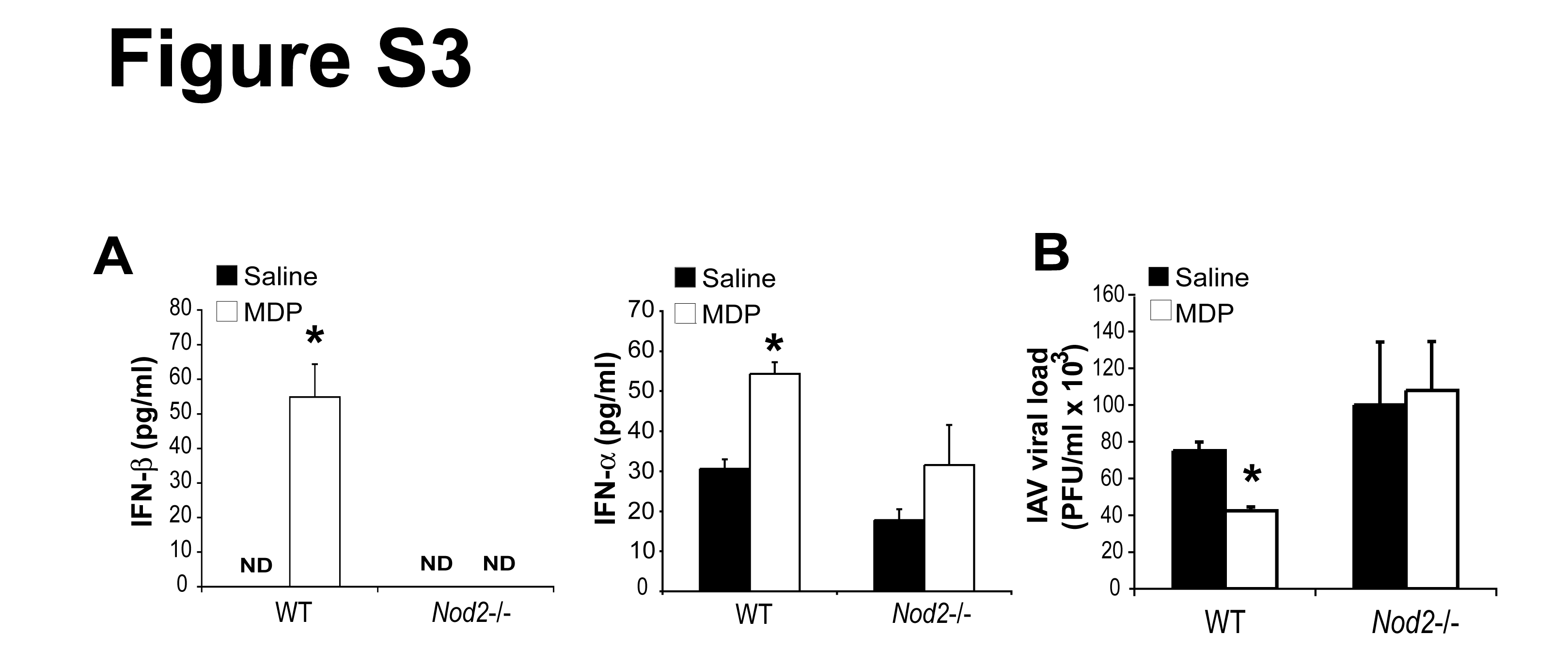

Supplement: Figure S3 — (A–B) WT and Nod2-/- mice (n = 3/group) were infected and treated daily with either saline or MDP (iv.). At day 3 pi., total lung cells from infected and treated WT and Nod2-/- mice were cultured ex vivo. Adherent cells were re-infected with IAV (1 MOI) for 16 hours. IFN-β and IFN-α (A) levels as well as viral loads (B) were determined from cell culture supernatants. Data shown are representative of two independent experiments (*p≤0.05 as compared to saline-treated mice, t-test). (TIF) [file pone.0036734.s003.tif]

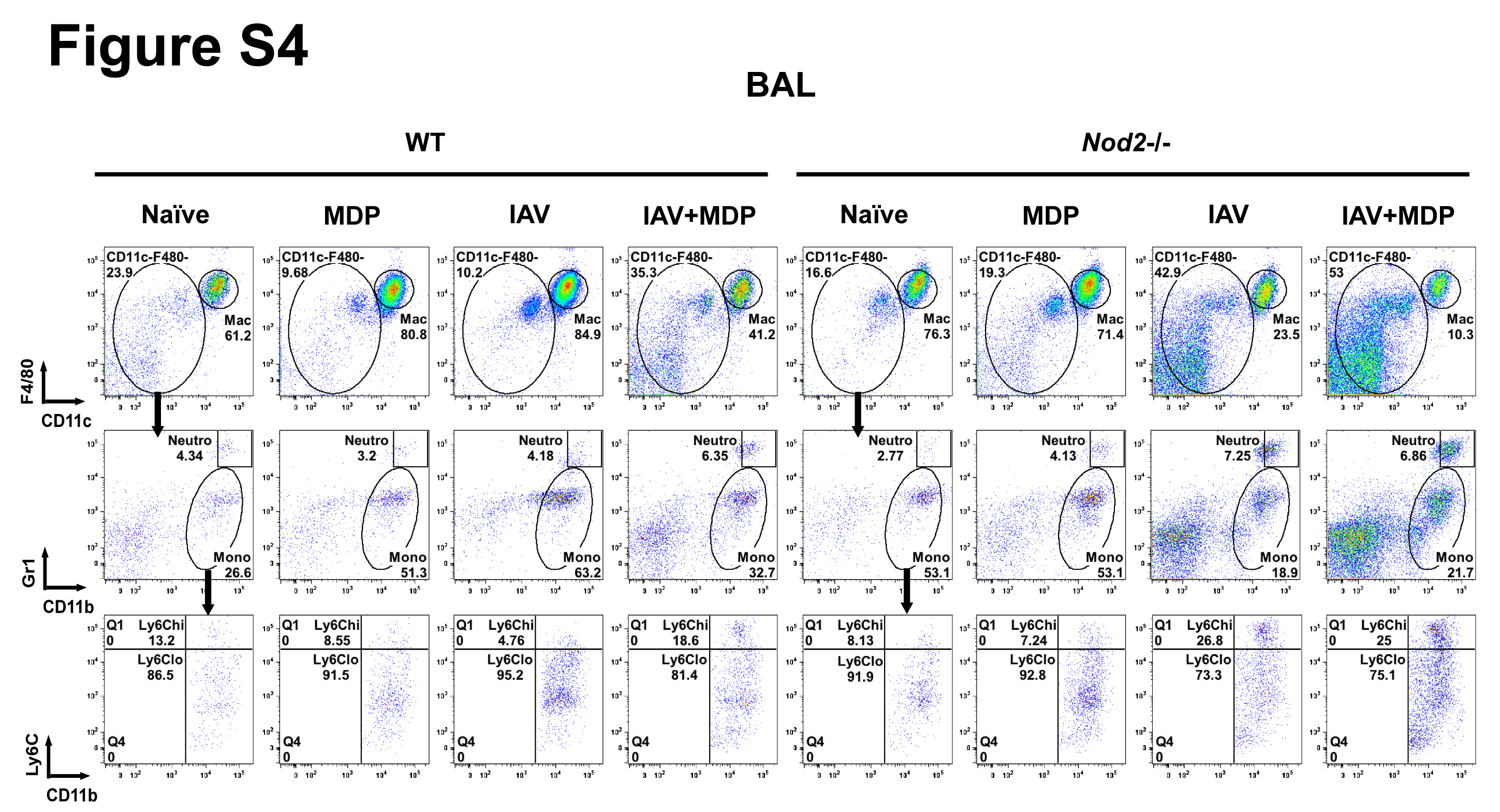

Supplement: Figure S4 — WT and Nod2-/- mice (n = 3/group) were infected or not with IAV and treated daily with either saline or MDP (iv.). At day 3 pi., BAL cells were subjected to flow cytometry analysis. Mac: macrophages; Mono: monocytes; N: neutrophils. Numbers indicate cell population percentages within gates. Data shown are representative of three independent experiments. (TIF) [file pone.0036734.s004.tif]
